# “Role of the B-cell receptor and the microenvironment in chronic lymphocytic leukemia''

**DOI:** 10.1038/bcj.2013.45

**Published:** 2013-09-20

**Authors:** P Oppezzo, G Dighiero

**Affiliations:** 1 Unit of Recombinant Protein, Institut Pasteur de Montevideo, Montevideo, Uruguay; 2 Immunobiology Department, School of Medicine, Universidad de la República, Montevideo, Uruguay; 3 Institut Pasteur de Montevideo, Montevideo, Uruguay; 4 Service of Hematology and Bone Marrow Transplantation, Hospital Maciel, Montevideo, Uruguay

**Keywords:** chronic lymphocytic leukemia, B-cell receptor, microenvironment

## Abstract

Despite significant progress in treatment, chronic lymphocytic leukemia (CLL) remains an incurable disease. Advances have been made to understand the molecular pathogenesis underlying CLL progression and treatment resistance. We here review the available evidences concerning the role of the B-cell receptor (BCR) and the tumor microenvironment interactions in CLL pathogenesis. Antigen likely has a key role in the selection of the tumoral clone, the mutational status of immunoglobulin genes is a strong prognostic predictor and BCR signaling has been postulated to have a role for CLL trafficking and interaction with the stromal microenvironment. There is also important evidence, favoring a role for the microenvironment in CLL pathogenesis. Most, if not all, proliferative events occur in the lymph nodes and bone marrow, where leukemic cells receive through microenvironment interactions survival signals aiming to avoid apoptosis and acquire favorable tumoral growing conditions. In addition, the tumoral microenvironment appears to be the site where the acquisition of additional genetic lesions in the clone occur, which should greatly influence clinical outcome. The advent of new tyrosine kinase inhibitors which seem to be able to modulate microenvironment interactions and circumvent the p53 deletion have generated significant promise by raising the possibility that they could provide significant progress in disease treatment.

## Introduction

Chronic lymphocytic leukemia (CLL) is the commonest form of leukemia in Western countries and mainly affects elderly individuals. It follows an extremely variable course, with survival ranging from months to decades.^1^ This leukemia is characterized by the accumulation of long-lived circulating clonal leukemic B cells resulting from a complex balance between cell proliferation and apoptotic death. Most tumor cells are arrested in cell cycle G0/G1 stages and only a minimal portion of the clone displays a proliferative activity.^2^ Available treatments often induce remissions, although almost all patients relapse, and CLL remains an incurable disease.

Recently, significant progress has been made in our understanding of CLL pathogenesis. CLL has traditionally been considered as an accumulative disease deriving from an inherent defect in apoptosis or in their cell death program. However, recent studies showed that B-CLL is a dynamic process composed of cells that also proliferate and die, often at appreciable levels.^3^ Using a non-radioactive, stable isotopic labeling method to measure CLL kinetics, Messmer *et al.*^4^ demonstrated that B-CLL is not a static disease that results simply from the accumulation of long-lived lymphocytes, but a disease in which coexist a proliferative and an accumulative pool. In these conditions, the evolution of the disease probably depends on the relative importance of these pools.

Important advances in the molecular pathogenesis were obtained through the study of the role of the B-cell receptor (BCR) and the microenvironment. In this review, we have tried to discuss, on the basis of our previous work and available evidence the role of the BCR and microenvironment in CLL evolution.

The BCR is an essential signal transduction pathway for the survival and proliferation of mature B lymphocytes and likely has a major role in the context of prognosis and positive selection of the precursor tumoral cell. Evidence for the important role of the BCR in CLL pathogenesis is given by the fact that the mutational status observed in the BCR sequences is one of the strongest predictors of disease outcome.^5, 6^ In addition, it constitutes a foundational event in CLL ethiopathogenesis, as suggested by the fact that 20% of CLL cases from unrelated patients display extremely similar, sometimes even identical antigen receptors. In addition, BCR signaling has been postulated to have a role for CLL trafficking and interaction with the stromal microenvironment.^7^ However, since our initial publication,^8^ where we succeeded to show that there was a 10-fold decrease in the expression of membrane Igs, we recently could demonstrate that this poor expression was the consequence of an important defect in the assembly of the BCR.^9^ In addition, it is clear that when stimulated through the BCR pathway, the response of CLL cells is impaired. Low expression of the BCR correlates with reduced induction of protein tyrosine kinase activity and defective intracellular calcium mobilization and tyrosine phosphorylation.

Although the possibility that BCR signaling has an important role in CLL evolution and progression, although this possibility cannot be excluded, concerns can be raised. Available evidence exists suggesting that the interaction of leukemic cells with stromal and T cells in the microenvironment has a key role in CLL evolution. It is clear that most, if not all, proliferative events occur in the lymph nodes (LN) and bone marrow, where leukemic cells are able to exploit microenvironment interactions with stromal cells and T-lymphocytes, in order to avoid apoptosis and acquire favorable tumoral growing conditions. As there is increasing evidence that individual cancer samples are heterogeneous and include subclonal populations and that tumors likely evolve through competition and interaction between different subclones, this tumoral microenvironment appears to be the site where acquisition of additional genetic lesions in the clone occur, which, should greatly influence clinical outcome. In line with this hypothesis, the LN CLL B-cells were found to show increased proliferation when compared with peripheral blood (PB) cells and were found to have a gene expression profile compatible with activated B cells.^10^

During recent years, a variety of novel kinase inhibitors aiming to target various components of the BCR signaling pathway have been designed. These mainly include phosphoinoside 3́kinase (PI3K)^11^ and Bruton tyrosine kinase (BTK).^12^ These drugs share a unique pattern of response resulting in nodal reduction and increased PB lymphocytosis. This likely reflects microenvironment modulation, which could lead proliferative leukemic cells, by an, as yet, unknown mechanism, to abandon protective microenvironment niches and thus induce them into apoptosis. In addition, these drugs are better tolerated than traditional chemotherapies and might be efficacious in the setting of traditional high-risk prognostic factors such del(17p). Thus, these new drugs targeting the signalosome have generated considerable excitement and could have a major role in new treatment strategies aiming at curing disease, as they could target the proliferative pool.

## The BCR in CLL

The BCR is a multimeric complex formed by the assembly of surface immunoglobulin (Ig) homodimer and the noncovalently bound heterodimer Igα/Igβ (CD79a/CD79b). This latter has a key role for receptor expression and signal transduction, by linking the antigen-binding Ig chains to intracellular tyrosine kinases of the Src-family.^8^ The BCR pathway is used by normal B cells to promote cell proliferation and to induce antibody production. Once encountering the antigen, the activated BCR recruits kinases such as spleen tyrosine kinase (SYK) and LYN, which phosphorylate the immunoreceptor tyrosine-based motifs receptors of Igα/Igβ.^13^ Phosphorylation of these induces a cascade of downstream events, which include that activation of BTK and PI3K,^14^ which subsequently induce the mobilization and activation of other downstream kinases such as protein kinase C-β, mammalian target of rapamycin and mitogen-activated protein kinase ERK.^15^ The activation of this cascade promotes survival and proliferation of B cells through the upregulation of transcription factors such as nuclear factor κ β (NF-κβ).^16^

The BCR is critical for normal B lymphocyte survival and is retained in the majority of B-cell malignancies. Thus, it constitutes a privileged marker as the BCR has unique Ig rearrangements that characterize the tumor cell and reveal the nature of its B-cell origin. Three main phenotypic features define the CLL B cell: (a) the predominant population shares B-cell markers (CD19, CD20 and CD23) with the CD5 antigen, in the absence of other pan-T-cell markers; (b) the B cells are monoclonal with regard to expression of either κ or λ light chains; and (c) express surface immunoglobulin, CD79β, CD20 and CD22 with low density. These characteristics are generally adequate for a precise diagnosis of CLL, and they also distinguish CLL from other disorders such as prolymphocytic leukemia, hairy-cell leukemia, mantle-cell lymphoma and other lymphomas.^17^

**Figure 1:**
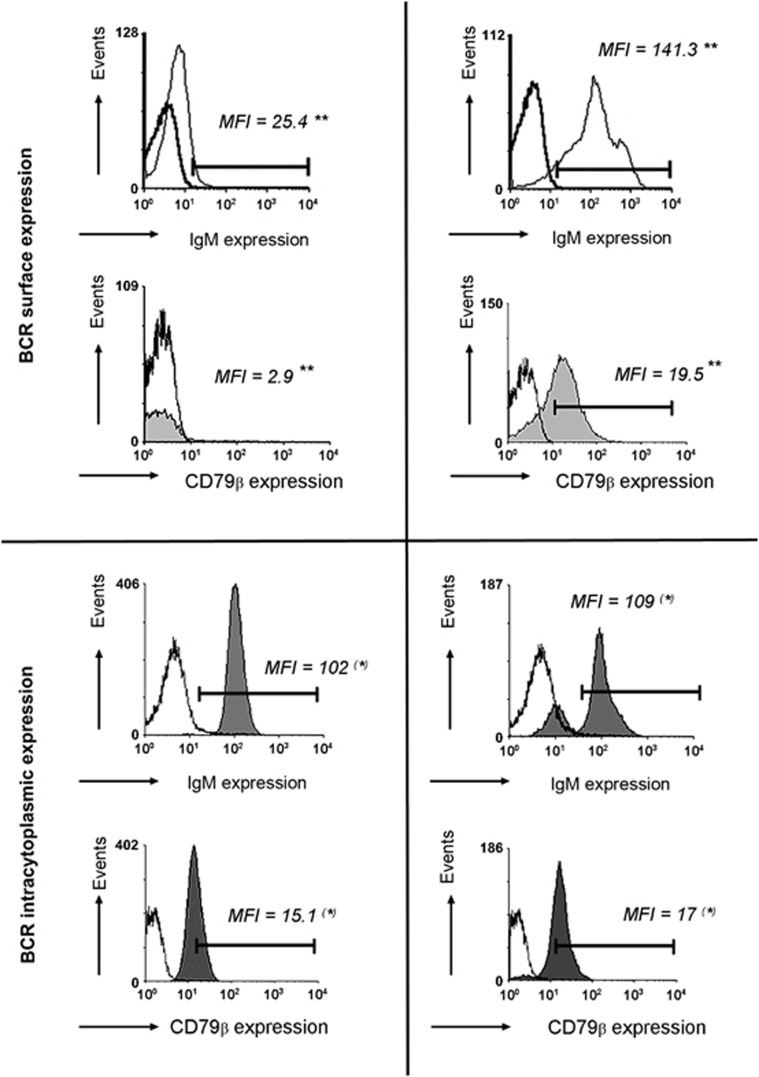
Flow cytometry analysis of surface and intracellular BCR molecules. Results from a representative CLL UM case and from a representative healthy subject are shown. Preparations of PB enriched CLL and normal B-cells by CD19 isolation, were subjected to flow cytometry, comparing isotype control staining (bold line), anti-μ-specific monoclonal antibody (mAb) (thin line) and anti-CD79β (gray plot). Fluorescence intensities are shown on a logarithmic scale on the axis and mean fluorescence intensity (MFI) is depicted. Statistically significant differences (**) are found regarding BCR surface expression and not statistically significant differences (*) when BCR intracytoplasmic expression is analyzed.

Low expression of the BCR is the hallmark of the B-CLL lymphocyte and is unique to CLL among mature B-cell malignancies. The mechanisms accounting for poor expression of the BCR in CLL remain elusive. Despite one single report,^18^ there is consensus on the absence of genetic defects in the BCR components.^19^ Although there are individual differences among patients, almost all CLL cases display very low levels of surface IgM and CD79β, when compared with normal B cells. In contrast with their poor expression at the membrane level, the mRNA transcription as well as intracellular synthesis of BCR components appear to be normal^20, 21^ (Figure 1).

For proteins bearing several subunits, such as the BCR, a correct folding and a proper assembly are necessary. These processes take place in the endoplasmic reticulum (ER) where proteins are modified (cleavage of signal peptide, N-glycosylation, formation of disulfide bonds) and then properly folded before exit to the Golgi. If the folding and maturation process fails, several different mechanisms are used by the quality control system of the cell to avoid the production of non-native proteins. The proteins are retained in the ER and exposed to resident chaperones to attempt adequate folding. In the case of BCR, several chaperones such as calnexin, calreticulin, glucose-regulated protein 78 also known as BiP (Ig heavy chain-binding protein) and GRP94 have been shown to associate with μ CD79α and CD79β chains.^22^

**Figure 2:**
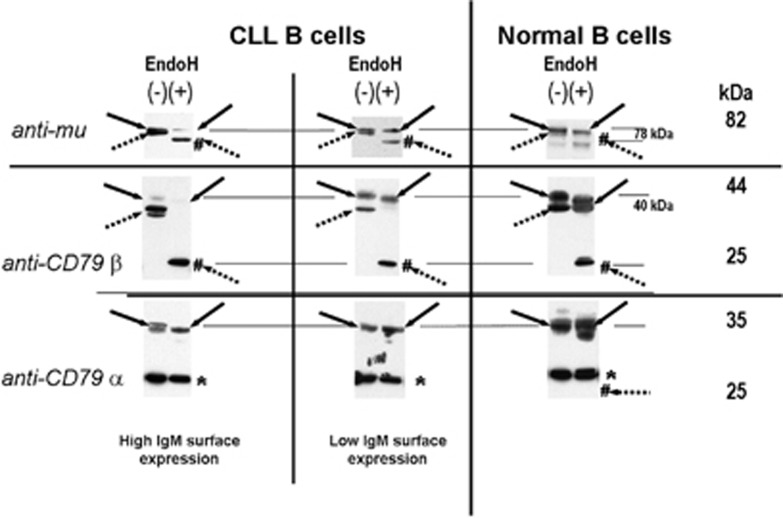
BCR component glycosylation status between CLL patients and healthy subjects. Cell extracts (20 ug) produced by lysis were incubated at 37 °C in the presence (+) or absence (−) of Endo-H. SDS-polyacrylamide gel electrophoresis and western blot were performed in order to visualize the different BCR components. Nitrocellulose membrane were probed with mAbs as follows: rabbit anti-heavy μ-chain, mouse anti-CD79α or anti-CD79β and immuno reactive bands were detected with an appropriate horseradish peroxidase-linked secondary antibody. Immature glycosylated (dotted arrows) and mature glycosylated (solid arrows) proteins are indicated for each staining. Deglycosylated forms by Endo-H treatment are indicated by #. Molecular masses are indicated in kilodaltons (kDa). The asterisk indicates a non specific band at 30 kDa constantly observed with the anti-CD79β probe from Pharmingen (Groningen, The Netherlands).

In a previous work,^23^ we found that nascent IgM failed to be processed and transported to the membrane. To gain insight into the relationship between this defect and IgM surface expression, we analyzed the different components of the BCR for their glycosylation status in 10 CLL patients. Six CLL cases displaying very low expression (patients no. 1–6), and higher, although significantly lower than normal, for patients no. 7–10 individual differences concerning surface IgM expression. Figure 2 depicts the differential pattern of IgM expression and glycosylation status of BCR components in two representative CLL subjects (one with very low IgM surface expression and the other with higher expression) versus normal B cells. In normal B cells, there was predominance of the mature μ chains of 82 kDa and the low-molecular weight form of 78 kDa accounted for a minority (Figure 2, panel μ, lane 5). Immunoblot analysis performed with CD79α monoclonal antibody in normal B cells indicated reactivity at 44 (mature form) and 40 kDa (immature form) (Figure 2, panel CD79a, lane 5). With regard to patient with higher IgM surface expression (CLL no. 08, Figure 2) as compared with patient no. 01 with the weakest IgM surface expression, bands of 82 kDa μ chain and 44 kDa CD79α chain were still detected in patient no. 08 as compared with very low expression of these molecules for patient 01 (panels μ and CD79α, lanes 1 and 3). Surprisingly, no difference was visualized between the CLL patients and normal control on membranes probed for the presence of CD79β (data not shown). Among the 10 patients studied, 6 were displaying a striking predominance of these low-molecular weight forms of μ and CD79α and roughly follows the pattern observed for the patient 01 depicted in Figure 2. Four out of six were indolent mutated forms of CLL (patients no. 1–4), one was mutated progressive stage B (patient no. 5) and one unmutated (UM) progressive stage A (patient no. 6). The four other patients displayed a mixed profile including both mature and immature μ and CD79a chains, which was closer to the normal pattern but still displayed a significantly higher amount of immature forms (78 kD for μ and 40 kD for CD79α). These patients corresponded to progressive forms of the disease, three were UM (patients no. 08–10) and one mutated (patient no. 07), and roughly follows the pattern observed for the patient depicted in Figure 2.

Next, experiments were conducted to determine whether these differences between CLL patients could reflect the presence of variably glycosylated forms of the different BCR components by treating cell extracts with Endo-H. This latter cleaves high mannose oligosaccharides associated to ER-localized glycoproteins, whereas resistance to enzymatic digestion is acquired after transport to the medial Golgi. Thus, Endo-H allows to discriminate ER-resident (immature) from Golgi-processed (mature) glycoforms.^9^ With normal cell extract, only a minority of μ and CD79α chains shifted to, respectively, 70 kDa and 25 kDa bands corresponding to immature glycoforms (Figure 2, panels μ and CD79a, lanes 5 and 6). In contrast, a majority of μ and CD79α chains corresponded to immature glycoforms in patients from group 1 (Figure 2, panels μ and CD79α, lanes 1 and 2), whereas more mature glycoforms, although to a lesser extent than in normal B cells, were found for group 2 (Figure 2, panels μ and CD79α, lanes 3 and 4). Interestingly, complete resistance to Endo-H was found for CD79b molecules from normal and all CLL cases (data not shown) indicating that this molecule has transited through the Golgi apparatus. Altogether, the results clearly indicate that the level of IgM surface expression in CLL is directly related to the level of glycosylation pattern impairment of μ and CD79α chains.

**Figure 3:**
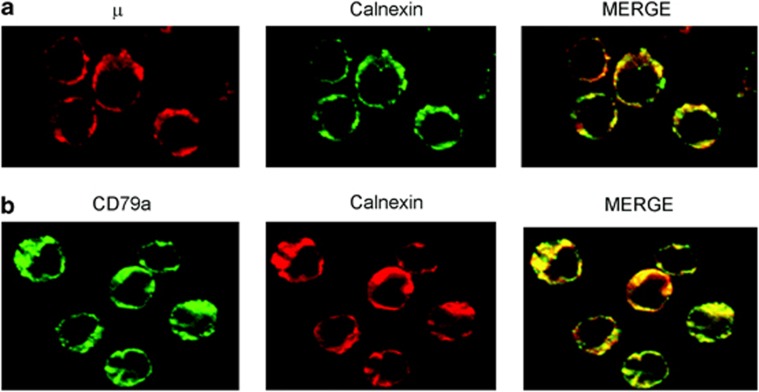
Analysis of the subcellular localization of the BCR components by immunofluorescence microscopy. Fixed and permeabilized CLL B cells were incubated with combinations of mAbs as follows: anti-μ/anti-calnexin (**a**), anti-CD79α/anti-calnexin (**b**). Red and green images were collected and merged with yellow coloration indicating colocalization.

As a consequence of their glycosylation defect and inability to assemble, cytoplasmic BCR components are retained in the ER compartment by the chaperone protein calnexin and cannot be transported from to the cell surface. Figures 3a, b shows that both μ and CD79α molecules are retained in the ER compartment by its resident chaperone protein calnexin.

A recent study, confined to surface Ig confirmed this glycosylation defect and demonstrated that a percentage of immature glycoforms could, however, reach the surface and postulated that these immature Ig glycoforms that predominate among UM cases could provide tonic survival signals.^24^ Nevertheless, as the membrane expression of surface immunoglobulin is very poor in CLL, this only concerns a minority of the expressed intracellular Igs.

Most B-CLL cells express CD5 and IgM/IgD and thus have a mantle zone-like phenotype of naive cells, which, in normal conditions express UM Ig genes.^17^ However, 50–70% of CLL harbor somatic mutations of *IgVH* genes^25^ as if they had matured in a lymphoid follicle. Although the origins of the CLL leukemic clone remains unsolved,^26,27^ the presence or absence of somatic mutations is associated with the use of particular *IgVH* genes.^28^ Two reports demonstrated that the clinical behavior of CLL is related to the mutational status of *Ig* genes. Patients whose B cells express mutated VH genes have a more indolent disease and longer overall survival than do patients expressing UM genes.^5, 6^ The mutational profile of *Ig* genes delineates prognostic groups within all Binet's stages.^1^ Despite this important difference in terms of prognosis gene expression profiling identified mutated and UM CLLs as part of a shared disease process with a common gene expression signature.^29,30^

The analysis of IgVH repertoire in CLL has demonstrated biases both at the family and specific gene segment usage.^31^ In recent years, several groups have reported differences in *IgVH* gene usage in CLL among different geographic areas (Europe, USA, China, Japan and Iran).^32^ Also, somatic mutations are not uniformly distributed within *IgVH* gene families, as they predominate among *IgVH3* and *IgVH4* families, whereas an UM profile is prevalent in the *IgVH1* family. The most frequently used *IgVH* genes in CLL rearrangements in Western countries are *IGHV1–69, IGHV3–07, IgVH 3–21* and *IgVH 4–34*.^32^ However, the available data from Japan, China and Iran show some differences with those from Western countries. The frequency of *VH1–69* in Japan and Iran is lower than that in Western countries. In addition, the frequency of the *VH 3–21* gene usage appears to be higher in Northern European countries when compared with Mediterranean ones. Evidence for the notion that CLL is a tumor of antigen-experienced B cells comes from the structure of the rearranged *IgV* genes. Analyses of large panels of CLL cases revealed that certain *IgV* gene family members, which could be hypermutated or UM, were expressed significantly more frequently in CLL than would be expected from their expression in the *IgV* gene repertoire of normal B cells.^28^ Although there is evidence favoring the idea that BCR stimulation by the antigen could have an important role in CLL evolution, concerns have been raised against this possibility. To formally prove this hypothesis, we should be able to proceed to stimulation of CLL cells with the antigen recognized by the BCR. Unfortunately, this putative antigen remains unknown.

## Role of the microenvironment in CLL evolution

CLL can be defined as a low-grade CD5+ B-cell tumor, whose tumoral cells have previously encountered the antigen, escaped programmed cell death and undergone cell cycle arrest in the G0/G1 phase. In CLL cells, elevated levels of the cyclin-negative regulator p27-Kip1 protein are found in a majority of patients.^33^ Given the key role of this protein in cell cycle progression, its overexpression in CLL cells may account for the accumulation of B cells in early phases of the cell cycle.^17^ In addition, overexpression of the anti-apoptotic BCL-2, BCL-XL, BAG-1 and MCL-1 molecules and the absence of microRNAs miR-15 and miR-16,^34^ whereas proapoptotic proteins like BAX and BCL-XS are under expressed^35^ could explain the resistance of tumoral cells to apoptosis. Despite the fact that most leukemic cells are arrested in cell cycle G0/G1 stages, Messmer *et al.*^4^ demonstrated that CLL is not only a static disease but also a disease in which a proliferative pool coexist. In consequence, evolution of this leukemia could depend on the relative balance between these subpopulations.

In contrast with *in vivo* results, apoptosis occurs after *in vitro* culture, suggesting a role of the microenvironment in CLL cell survival.^36^ Within the leukemic microenvironment, two cellular components appear to be potential players: stromal cells and T-lymphocytes. *In vitro,* the spontaneous apoptosis of B-CLL can be rescued by stimulation via surface CD40 and interleukin-4,^37^ by the coculture with mesenchymal stromal cells^38^ and/or ‘nurse-like cells'.^39^

CLL cells seem to recruit accessory cells;^40^^41^ and thereby create a microenvironment that supports their own survival. There is an increase of CD3+ T cells, most of which are CD40L+CD4+, which cluster in and around pseudofollicles. These cells can stimulate CLL cells through the interaction of CD40 and CD40L, and this stimulus synergizes with BCR signaling.^42^ In addition, evidences exist that CD40–CD40L engagement activates NF-κβ/Rel transcription factors as well as Janus-activated kinase (JAK 3) and signal transducer and activator of transcription 3 to induce high levels of the anti-apoptotic proteins BCL-XL and MCL-1.^43^ NF-κβ activation, in turn, leads to upregulation of the TP63 which also acts through the integrin very late antigen-4 (CD49d) to facilitate the migration of CLL cells into the supportive microenvironments.^44^ When cocultured with CLL cells, mesenchymal stromal cells appears to be able to protect the neoplastic B cells from apoptosis induced by fludarabine and bendamustine.^45^ In turn, nurse-like cells are also able to protect CLL cells from apoptosis.^41,46^ Nurse-like cells secrete stromal cell-derived factor 1 and the tumor necrosis factor family ligands APRIL (a proliferation-inducing ligand) and BAFF (B-cell-activating factor), which protect CLL cells from apoptosis.^47^ Both mesenchymal stromal cells and nurse-like cells therefore have anti-apoptotic activity and the combination of these two cell types provides a supportive microenvironment for tumor cells in CLL.^48^

Lymphocyte trafficking between the blood and secondary lymphoid tissues is organized by tissue-specific expression of chemokines and their receptors on lymphocytes, which cooperate with adhesion molecules and their ligands. B-cell positioning in these areas is coordinated by the chemokines CXCL12 and CXL13, which are expressed by stromal cells,^49^ and CXCR4 and CXCR5, which are differentially expressed by the B cells.^50^ In addition, bone marrow and LN stromal cells secrete CXCL9,10,11,12,13 and CCL19 and 21 which bind CXCR3, CXCR4 and CXCR5 receptors different expressed by the leukemic clone.^51^ In turn, CLL B cells, also secrete chemokines such as CCL3, CCL4, CCL22 and interleukin-8, which can attract accessory cells, such as T cells and monocytes. This finding suggests that CLL cells are not simply seed in a supportive soil but instead are actively involved in a complex crosstalk that possibly will maintains a proliferative behavior.

Evidence indicates that the relationship of CLL B cells with their microenvironment is crucial for the creation of altered key proliferative/apoptotic pathways, which are hallmarks features in CLL pathogenesis. In a recent work, we have investigated the mechanisms accounting for the aberrant expression of the lipoprotein lipase enzyme in UM CLL patients.^52^ Our results, demonstrated that this aberrant expression resulted from the lack of methylation in the lipoprotein lipase CpG island. Interestingly, this epigenetic mechanism appeared to be mainly triggered by T-cell-dependent microenvironments signals (CD40L+interleukin-4), whereas cross-linking of the BCR poorly induced this demethylation process, and signaling through TLR9 or TLR1/2 pathways were found unable to induce it.

At this time, it is clear that crosstalk with accessory cells in specialized tissue microenvironments favors disease progression by promoting malignant B-cell growth and the emergence of new genetic alterations, which will lead to drug resistance.^41^ Therefore, understanding the crosstalk between malignant B-cells and their milieu could give us new keys on the cellular and molecular biology of CLL that can finally lead to novel strategies for disease treatment. In this regard, the isolation and analysis of the tumoral subset that is being triggered in the proliferative compartments of progressive CLL cases is an important aim to understand CLL pathogenesis. Different groups have tried to assess this question by studying different CLL proliferative subsets like that expressing CD38 marker,^53^ or that expressing activation induced cytidine deaminase (AID)^54^ or CD5/CXCL4 molecules.^55^

Under physiological conditions, the microenvironment stimulation occurs in the germinal centres and is triggered by the presence of an antigen. In this microenvironment, B lymphocytes undergo a series of specialized events resulting in the production of specific antibodies with both high affinity and adequate effector function. These processes are somatic hypermutation, in which different mutations are introduced into the variable domain of Igs and class switch recombination (CSR), which results in a change of antibody isotype. A useful humoral immunity is dependent on these two processes and both are absolutely dependent on AID enzyme.^56^ AID is expressed mainly in stimulated B cells and at the physiological levels is responsible for DNA damage in the pre-switch and variable VDJ regions that lead to DNA breaks and/or point mutations in the immunoglobulin genes.

**Figure 4:**
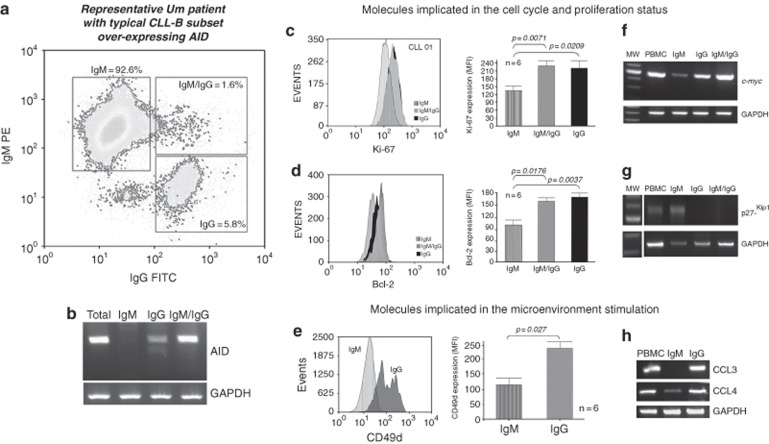
CLL B cells subset overexpressing AID enzyme is a hallmark of microenvironment stimulation and is associated to disease progression. (**a**) Representative flow cytometry assay from an UM AID^+^^+^ CLL patient. The three CLL B-cells, IgM^+^, IgM^+^/IgG^+^ and IgG^+^ fractions are depicted. (**b**) Semiquantitative reverse transcription PCR (RT-PCR) from the three cell sorter isolated subpopulations. (**c**–**e**) Flow cytometry analysis, in a representative UM CLL patient, showing Ki-67, Bcl-2 and CD49d protein expression, respectively. MFI means values (± s.e.) of Ki-67, Bcl2 and CD49 expression from six UM CLL patients with high AID expression. (**f**, **g**) p27-kip1 and c-myc semiquantitative RT-PCR from the cell sorter isolated subpopulations with specific primers. GAPDH was amplified in all cases as internal semiquantitative control. (**h**) CCL3 and CCL4 mRNA profile expression in the different isolated subsets (IgM^+^ and IgG^+^) from the same representative UM patient.

In our laboratory, we first reported that in contrast to normal circulating B lymphocytes, which only express AID transcripts following CD40L or LPS stimulation, in most CLL cases expressing UM VH genes, tumoral cells are able to express high levels of an active AID enzyme,^57^ although this expression is confined to a small proportion of the CLL clone.^58^ As AID expression results from signals mainly received through the CD40–CD40L pathway, we subsequently investigated whether its expression was related to CSR occurring in a small subset of CLL B-cells and whether it could have an increased proliferative potential. Our results showed that high AID expression is almost exclusively restricted to the subpopulation of tumoral cells having an ongoing CSR process and more important, the presence of this subpopulation in CLL is closely related to an aggressive course of the disease.^57, 59^ This small clonal subset having achieved CSR and expressing either IgG at the membrane or both IgM and IgG, displays higher levels of AID, and higher levels of proliferation and anti-apoptotic molecules like Ki-67, c-myc and Bcl-2. In addition, it also expresses high levels of proteins associated to progression such as CD49d and CCL3/CCL4 chemokines as well as a decreased expression of the cell cycle inhibitor p27-kip1 when compared with its quiescent tumoral counterpart expressing exclusively IgM (Figure 4).

Overexpression of AID has been linked to a loss of specificity on Ig genes and consequently to mutations and translocations in the typical lymphoma-associated oncogenes.^60^ In agreement with this, the overexpression of AID in transgenic animals leads to tumor development in several organs.^61^

It is accepted that AID expression is induced in germinal centres through the contact of T and B cells via CD40–CD40L interactions.^62^ However, it has been recently demonstrated that another costimulatory signals involving tumor necrosis factor-related factors such as BAFF (B-cell-activating factor) and APRIL (proliferation-inducing ligand)^47^ or TLRs (Toll-like receptors)^63^ and TLRs and BCR^64^ are also able to trigger AID expression and CSR. Thus, ongoing CSR and AID expression in the PB of UM progressive CLL cases appears to be a hallmark of a proliferative disease in which B lymphocytes are being constitutively activated in specific tumor microenvironments.^65^

A recent study compared gene expression profiling of PB to LN and bone marrow tumoral cells in 24 treatment naïve patients. This study identified LN as a key site for proliferation.^10^ CLL cells in the LN showed upregulation of gene signatures indicating BCR and NF-κβ activation. In addition, the expression of these genes was stronger in clinically more aggressive CLL and tumor proliferation as assessed by E2F, and c-MYC and Ki-67 expression was higher in LN and was correlated to disease progression. The authors concluded that these results could be useful to identify target molecules able to disrupt the tumoral microenvironment interactions and to inhibit the BCR signaling as promising therapeutic strategies in CLL.^10^^66^

**Figure 5:**
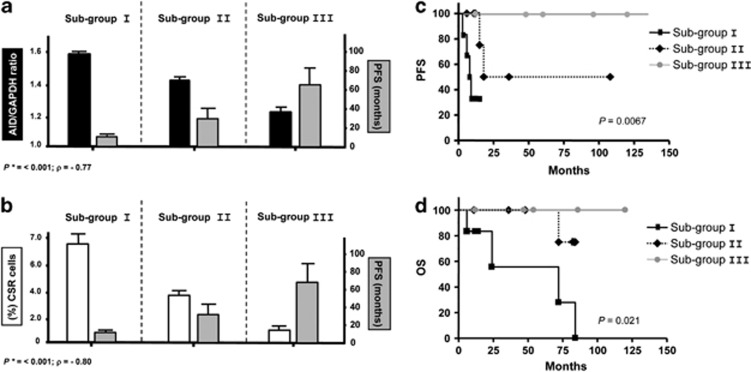
AID expression levels and high CSR segregate patients with UM CLL into three subgroups with different clinical progression. Subgroup I, corresponds to patients with high levels of AID expression and high clonal CSR (>5%), subgroup II to low AID expression and low clonal CSR (< 5%) and subgroup III to negative AID expression and non clonal CSR. (**a, b**) Correlations of AID expression and progression free survival (PFS) (**a**) and clonal CSR and PFS (**b**) for the three subgroups are plotted. Both analyses indicate a significant negative correlations; (P*) by Spearman rank test for AID expression correlated to PFS and for clonal CSR linked to PFS. (**c, d**) Kaplan–Meier curves based on AID and CSR expression in the three subgroups. The Kaplan–Meier method was used to construct survival curves for PFS (**c**) and overall survival (OS) (**d**). Results were compared with the log-rank test Spearman. *P*-values refer to the log-rank test.

The subset recently described by our group displays very similar markers to those found by Herishanu *et al.*^10^ in LN from CLL patients. This small subpopulation expressing AID in the PB of progressive UM CLL patients, probably results from the passage into the circulation of this proliferative pool occurring in LN. In agreement with this hypothesis, this pool can be identified in the PB of some UM CLL patients displaying a highly aggressive disease (Figure 5).

Being aware that AID overexpression is associated with the loss of target specificity resulting in mutations in non-immunoglobulin genes, (*BCL-6, MYC, PAX-5* and *RHOH)*,^61^ it is logical to assume that progressive disease could be related to clonal CLL evolution. In this regard, it has been also suggested that mutations in *TP53* genes could be related with AID expression in CLL.^67^ If true, the constitutive expression of AID in the leukemic clone history could be a key event in disease progression.

## Conclusions

CLL is characterized by an accumulation of monoclonal B lymphocytes expressing CD5 and CD23 molecules, but critical low amounts of surface Ig and CD79β molecules.^17^ Among B-cell malignancies, this phenotypic feature is unique to CLL and correlates with a defective tyrosine kinase activity resulting in reduced tyrosine phosphorylation upon stimulation through the BCR pathway, although a majority of UM cases (2/3), which exhibit a low response, appear to respond better than mutated ones.

Normal B cells following interaction with antigen-specific T cells or not in the case of T-independent responses should differentiate into plasma cells or memory cells. Those cells failing to be selected by their antigen affinity should be committed to death. In CLL, the tumoral process protects these cells from following apoptosis and allow them to survive without differentiation.

The BCR is an essential signal transduction pathway for the survival and proliferation of mature B lymphocytes and likely has a major role in the context of positive selection of the precursor tumoral cell. Thus, it constitutes a foundational event in CLL ethiopathogenesis and as suggested by the fact that 20% of CLL cases from unrelated patients can have extremely similar, sometimes even identical antigen receptors, this probably occurs as a consequence of a stringent selective process induced by the antigen. Antigen likely has an important role at one point during the ontogeny of CLL in the context of positive selection of the precursor cell. It is presently unclear whether it has a major role during the evolution of disease, as shown in other tumoral models like mouse lymphoma models or in some mucosa-associated lymphoid tissue lymphomas, where continuous antigen stimulation has a key role and antigen withdrawal leads to regression of disease.

During recent years, evidence accumulated suggesting that signaling via the BCR could have an important role in the development of CLL and that it could determine the variable clinical behavior of the disease. This could likely occur through antigen exposure *in vivo* which could induce signaling events for constitutive activation of kinases and of NF-κβ in CLL B cells.

The advent of new therapies that target the signalosome brings new evidence in favor of this view. Initial results from clinical trials with drugs that inhibit SYK kinase (fostamatimib), the BTK kinase (PCI-32765, Ibrutinib) and PI3K-δ (CAL-101, Adelalisib) are very encouraging.^68^ These drugs share a unique pattern of response resulting in nodal reduction and increased PB lymphocytosis likely reflecting that microenvironment modulation, which, could prevent these cells to receive the survival signals delivered by the microenvironment.

In addition, these drugs are better tolerated than traditional chemotherapies and might be efficacious in the setting of traditional high-risk prognostic factors such as del(17p). Thus, these new molecules targeting the ‘signalosome' have generated considerable excitement and could have a major role in new treatment strategies aiming at curing disease, as they could target the proliferative pool.

We previously described^23^ that transcription and intracellular synthesis of the BCR components were normal and that low BCR expression at the membrane resulted as a consequence of defective assembly of the different BCR components. Our results showed a severe impairment in glycosylation and folding of μ and CD79α chains, leading to their defective assembly and their retention in the ER compartment, which could account for the low levels of IgM surface expression in malignant B-CLL cells. Although a recent study confined to membrane Igs demonstrated that some of these immature glycoforms succeed to assemble and reach the membrane, the amount of Ig and particularly CD79α and CD79β that normally reach the membrane is very low and the majority of Ig produced by the cell remains retained in the reticulum endoplasmic compartment.

Moreover, even if we accept that UM cases respond better to stimulation through the BCR than the mutated ones, the response is poor in the vast majority of cases and at least one-third of these cases display very low responses to this stimulation.

Although promising, it may be premature to ascribe positive clinical responses of SYK, BTK and PI3k-δ inhibitors to specific inhibition of BCR-mediated signaling. In particular, these kinases also contribute to other signaling pathways independent of the BCR. For instance, in CLL, SYK phosphorylation is increased in cells stimulated via chemokine receptors and integrins and SYK inhibition reduced migration toward CXCL12 and adhesion to VCAM-1. The PI3K inhibitor CAL-101 also interferes with survival effects of CD40L, tumor necrosis factor-α and fibronectin in CLL cells and BTK is required for Toll-like receptor signaling.^64^ Thus, the contribution of specific BCR signaling inhibition to the clinical efficacy of these agents remains unclear as yet. Indeed, it is possible that their clinical effects stem from simultaneous inhibition of multiple signaling responses.

There is increasing evidence that individual cancer samples are heterogeneous and include subclonal populations and that tumors likely evolve through competition and interaction between different subclones. In the case of CLL, this so called proliferative pool may be the site where acquisition of additional genetic lesions in the clone occur. AID expression is induced through the contact of T and B cells via CD40–CD40L and other interactions, which results from signals received in this particular microenvironment. Our data indicate that the proliferative subset expressing AID and proliferative markers in PB, is the consequence of microenvironment interactions occurring in the specific proliferative CLL centres. As this subset expresses very similar markers to those found by Herishanu *et al.*^10^ in the LN, we can speculate that these cells result from the passage into the circulation of the proliferative pool occurring in the LN.

Overall, these new approaches have allowed better understanding of CLL pathophysiology and provided a more effective and rationale criteria for the management of this disease. How do these data help defining treatment in CLL?

Current chemotherapy options are able to obtain complete and long remissions, although relapse ineluctably occurs and a second round of chemotherapy usually results in inferior response. This probably results because we are presently unable to eliminate residual malignant clones located at the LN and bone marrow, which through their interaction with stromal and T cells, receive survival signals and are able to produce new genetic lesions like the 17p or 11q deletion.

Recent results pointing to an important role of BCR and microenvironment signaling in tumor progression may redirect therapeutic options to treatment aiming to selectively attack the proliferative pool existing in this protective microenvironment.
